# p21-Activated Kinases Are Required for Transformation in a Cell-Based Model of Neurofibromatosis Type 2

**DOI:** 10.1371/journal.pone.0013791

**Published:** 2010-11-02

**Authors:** Hoi Yee Chow, Dina Stepanova, Jennifer Koch, Jonathan Chernoff

**Affiliations:** Cancer Biology Program, Fox Chase Cancer Center, Philadelphia, Pennsylvania, United States of America; Calypte Biomedical Corporation, United States of America

## Abstract

**Background:**

NF2 is an autosomal dominant disease characterized by development of bilateral vestibular schwannomas and other benign tumors in central nervous system. Loss of the NF2 gene product, Merlin, leads to aberrant Schwann cell proliferation, motility, and survival, but the mechanisms by which this tumor suppressor functions remain unclear. One well-defined target of Merlin is the group I family of p21-activated kinases, which are allosterically inhibited by Merlin and which, when activated, stimulate cell cycle progression, motility, and increased survival. Here, we examine the effect of Pak inhibition on cells with diminished Merlin function.

**Methodology/Principal Findings:**

Using a specific peptide inhibitor of group I Paks, we show that loss of Pak activity restores normal cell movement in cells lacking Merlin function. In addition, xenografts of such cells form fewer and smaller tumors than do cells without Pak inhibition. However, in tumors, loss of Pak activity does not reduce Erk or Akt activity, two signaling proteins that are thought to mediate Pak function in growth factor pathways.

**Conclusions/Significance:**

These results suggest that Pak functions in novel signaling pathways in NF2, and may serve as a useful therapeutic target in this disease.

## Introduction

Neurofibromatosis type 2 (NF2) is an autosomal dominant disorder characterized by the development of bilateral vestibular schwannomas and other benign tumors in central nervous system [1], [2]. While several mitogenic pathways are known to be upregulated in *NF2*-mutant cells, despite considerable effort, there is as yet no consensus as to how loss of the *NF2* tumor suppressor gene leads to schwannoma growth, nor are there effective medical therapies for this disorder.

The protein encoded by the *NF2* gene, Merlin, exhibits significant homology to Ezrin-Radixin-Moesin (ERM) proteins, sharing a FERM (Four-point one, Ezrin, Radixin, and Moesin) domain at the N-terminus followed by an alpha-helical segment. Merlin has a unique C-terminal domain lacking a binding region for F-actin that exists in all other ERM proteins [3]. Within the FERM domain, a seven amino-acid conserved sequence (termed the “Blue Box”), is important for Merlin functions. In Drosophila, deletion of this sequence (ΔBB) or substitution of polyalanine within this region (BBA) results in a dominant-negative form of the protein [4], most likely by disrupting intramolecular association between the N- and C-termini of Merlin [5]. This self-interaction can also be disrupted by phosphorylation of Merlin at residue serine 518, leading to a functionally inactive “open state” [6]. Merlin phosphorylation at this site is stimulated by Rac1 and Cdc42 GTPases via activation of their downstream effectors, p21-activated kinases (Paks) [7], [8].

Merlin is known to play an inhibitory role in Rac-mediated signaling [6]. NF2-deficient Schwannoma cells display aberrant membrane ruffling and concomitant hyperactivation of Rac and Pak1 [9], [10], [11]. Fibroblasts and keratinocytes lacking Merlin lose contact inhibition and *Nf2*-null neuroendocrine cells are defective in assembly of tight junctions and adherens junctions [12], processes disrupted by activated Rac and Pak [13], [14], [15]. Merlin affects Rac trafficking to the plasma membrane and is a direct inhibitor of Pak1, suggesting a negative feed-forward loop between Rac/Pak and Merlin [16]. For these reasons, The Rac/Pak signaling axis has garnered increasing attention as a possible therapeutic target in NF2.

As downstream mediators of Rac function, Paks have been implicated in regulating cell morphology, motility proliferation, and survival [17], [18], [19]. Group I Paks (Pak1, -2, and -3) affect a wide variety of central signaling pathways, including positively regulating mitogen activated kinases (MAPKs), Akt, and NFkB [20], [21]. In addition, group I Paks influence the G2/M transition by activating Aurora-A and Polo-like kinase 1 (Plk-1) [22], [23]. In Ras-transformed cells, expression of dominant- negative Pak1 blocks transformation and prevents full activation of Erk and Jnk, and it is thought such MAPKs represent important signaling targets for Pak in cancer [24], [25]. In the Erk pathway, Pak has been shown to phosphorylate c-Raf at S338 and Mek1 at S298 [26], [27], [28], [29]. These phosphorylations are thought to be necessary, but not sufficient, for full activation of c-Raf and Mek by Ras.

In this study, we show that inhibition of group I Paks in *Nf2*-mutant fibroblasts reduces proliferation, restores normal morphology, and reduces invasiveness of cells lacking Merlin function, as well as the tumorigenicity of such cells in nude mice. However, these changes are not associated with reductions in Erk or Akt activity; in fact, the activity of these pathways is augmented, suggesting that the beneficial effects of inhibiting Pak in these cells involves novel signal transduction pathways.

## Results

### Establishing Merlin-deficient cells that express a Pak inhibitor

**Figure 1:**
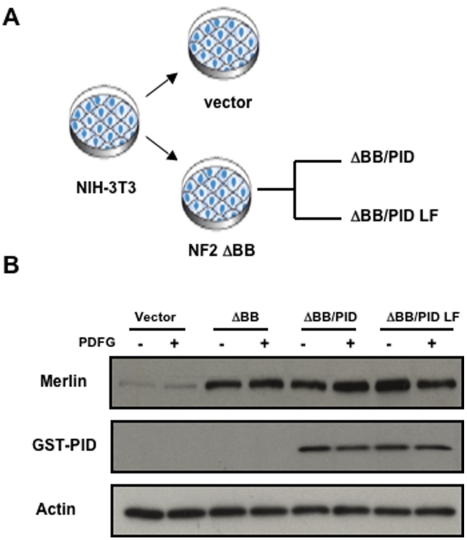
Establishment of cells with loss of Merlin and/or Pak function. (**A**) Schematic of experimental design. NIH-3T3 cells were infected with a control retrovirus or a retrovirus encoding dominant negative Merlin (Merlin ΔBB). These cells were then infected with a retrovirus encoding the Pak inhibitor (PID) or an inactive control (PID LF). (**B**) Cells were starved overnight and then stimulated with PDGF for 5 min. Immunoblots for Merlin, (GST)-PID, and actin are shown.

To study the influence of Pak inhibition in cells lacking Merlin function, we established a stable NIH-3T3 fibroblast cell line overexpressing Merlin ΔBB, a mutant form of the tumor suppressor that lacks the “Blue Box” motif and which, in *Drosophila*, acts in a dominant negative fashion, in effect mimicking loss of the NF2 gene (Fig. 1A) [30]. Another “Blue Box” mutant, Merlin BBA, has been shown to be tumorigenic in mammals [31], [32], though Merlin ΔBB and Merlin BBA are reported to show some differences with respect to effects on cell shape and adhesiveness [30], [31]. The expression level of Merlin ΔBB in such infected cells was about 5-fold higher than endogenous Merlin (Fig. 1B and Fig. S1). Note that, despite deletion of the Blue Box motif, exogenous Merlin ΔBB migrates at the same position as endogenous Merlin in SDS/PAGE. NIH-3T3 cells expressing dominant negative Merlin (ΔBB cells) were then infected with recombinant retrovirus encoding GST-tagged Pak inhibitor domain (PID) or a nonfunctional inhibitor control, PID L107F (PID LF) [27]. The ΔBB cells infected with the PID or PID LF retrovirus displayed readily detectable signals in anti-GST immunoblots (Fig. 1B).

### Effects of Pak inhibition on invasiveness in cells expressing dominant negative Merlin

**Figure 2:**
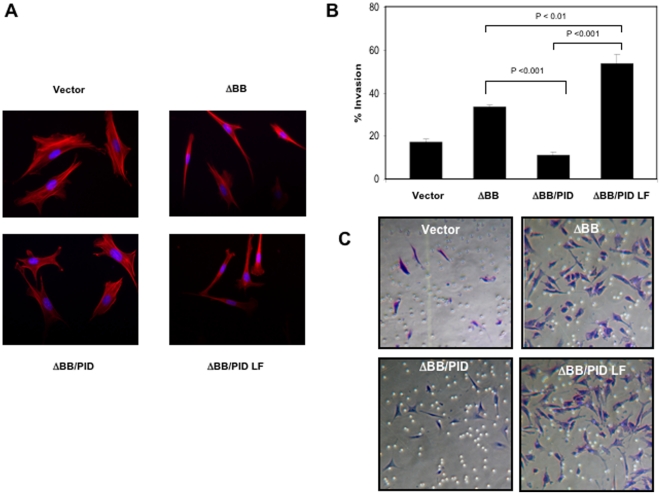
Effects of PID on morphology and invasion. (**A**) Cells expressing Merlin ΔBB, Merlin ΔBB/PID, or Merlin ΔBB/PID LF were fixed and stained for F-actin (red) and for DNA (blue). (**B**) The invasiveness of control cells or cells expressing Merlin ΔBB, Merlin ΔBB/PID, or Merlin ΔBB/PID LF was determined over 12 hr by Matrigel invasion chamber assay as compared against uninfected control cells. The experiments were performed three times with similar results. The data are presented as the means ± S.D. of duplicate well measurements from one representative experiment. (**C**) Staining pattern of cells that have migrated and adhered to the bottom surfaces of filters in cell invasion assay. The small circles are cross-sections of 8 µm pores. Scale bar  = 50 µm.

**Figure 3:**
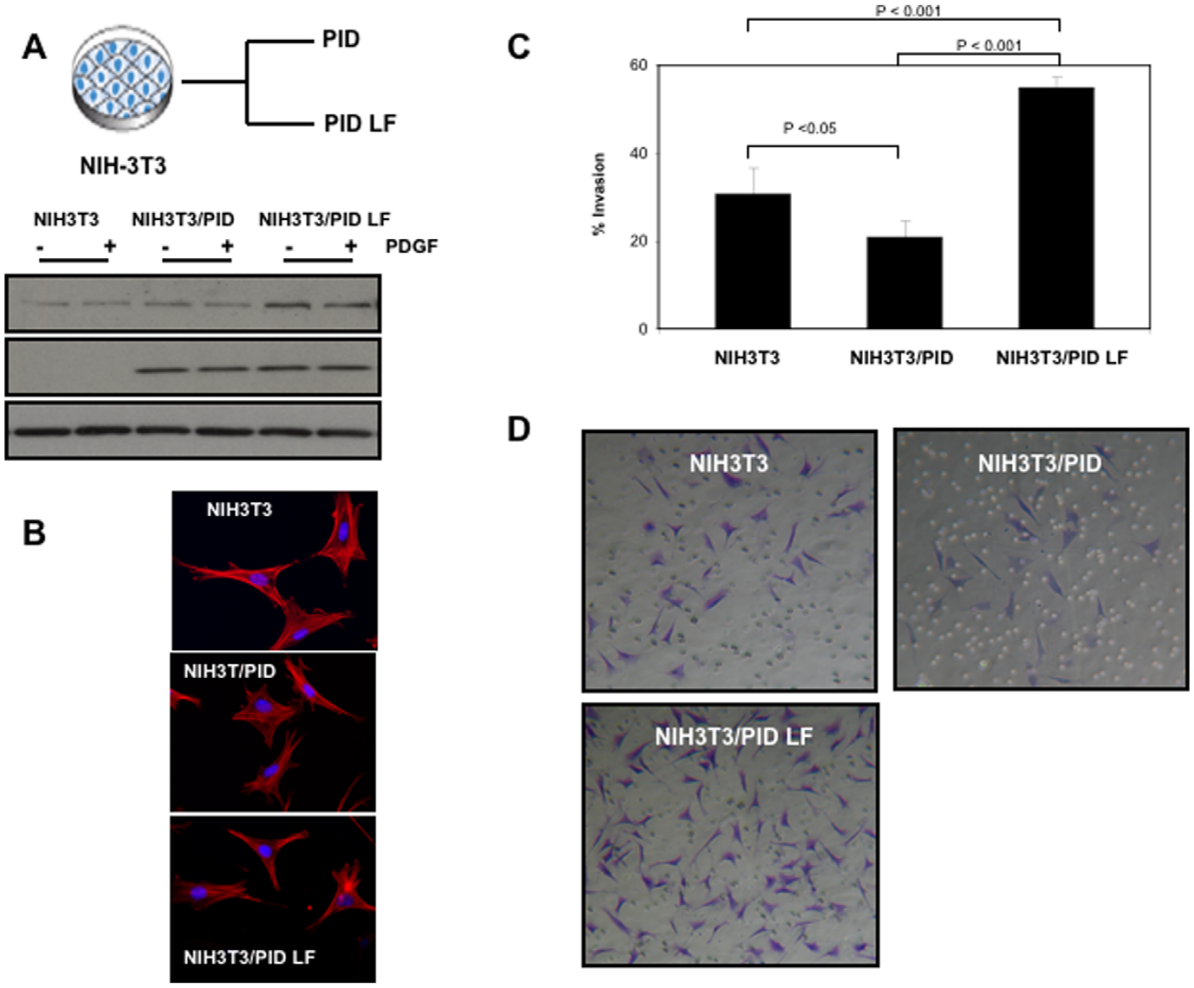
Effect of Pak inhibition on morphology and invasiveness in NIH-3T3 cells. Control NIH-3T3 cells and NIH-3T3 cells stably expressing PID or PID LF were starved for 24 hrs in serum-free medium, then stimulated for 5 min with 5 ng/ml; PDGF. (**A**) Protein lysates were separated by SDS-PAGE, blotted, and probed with the indicated antibodies. (**B**) Cells expressing Merlin ΔBB, Merlin ΔBB/PID, or Merlin ΔBB/PID LF were fixed and stained for F-actin (red) and for DNA (blue). (**C**) The invasiveness of control cells or cells expressing Merlin ΔBB, Merlin ΔBB/PID, or Merlin ΔBB/PID LF was determined over 24 hr by Matrigel invasion chamber assay as compared against uninfected control cells. The experiments were performed three times with similar results. The data are presented as the means ± S.D. of duplicate well measurements from one representative experiment. (**D**) Staining pattern of cells that have migrated and adhered to the bottom surfaces of filters in cell invasion assay. The small circles are cross-sections of 8 µm pores. Scale bar  = 50 µm.

We first examined the effect of Merlin on cell morphology and invasiveness and asked if such effects could be reversed by PID expression. Cells expressing ΔBB became fusiform as compared to normal NIH-3T3 cells (Fig. 2A). Expression of the PID, but not the control PID LF, showed substantial restoration of normal morphology. For invasion studies, cells were plated in a chamber above a layer of Matrigel and assessed for their ability to penetrate through this layer, indicating invasiveness. As shown in Fig. 2B and Table S1, the invasiveness of ΔBB cells was almost twice that of control NIH-3T3 cells. Assuming linear invasion kinetics, expression of PID, but not PID LF, significantly inhibited the invasive capacity of Merlin ΔBB cells. Thus, a Pak inhibitor substantially reduced invasiveness in cells expressing a dominant-negative form of Merlin. In control NIH-3T3 cells, PID expression had a slight effect on morphology, and reduced invasiveness by about 10% (Fig. 3 and Table S2).

### Inhibition of tumor formation by Pak inhibition

**Figure 4:**
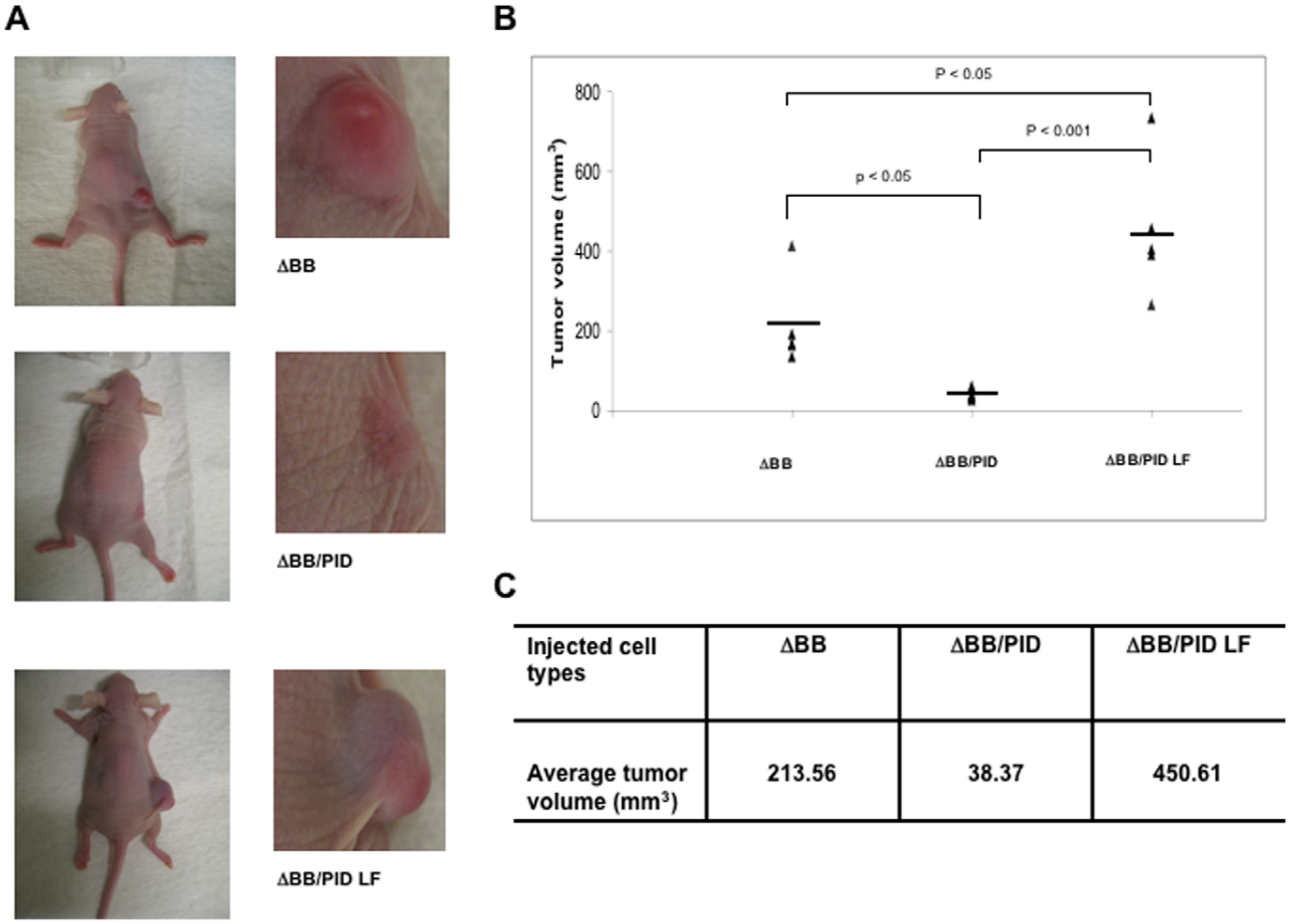
Effects of PID on tumor formation. Tumor formation was assessed by subcutaneously inoculating 1×10^6^ NIH-3T3 cells infected with empty vector (Vector) into left flanks and equal number of Merlin-expressing cells (ΔBB, ΔBB/PID and ΔBB/PID LF, respectively) into right flank of the same mice. Five mice were used for each cell line. Tumor diameters were measured every 3 days using caliper for 6 weeks following injection. (**A**) Representative photographs of nude mice with solid tumors (left panel). Enlarged images of boxed area in the left panel (right panel). (**B, C**) Tumor volume was calculated by the formula described in Material and Methods. Horizontal lines represent the average tumor diameter.

**Figure 5:**
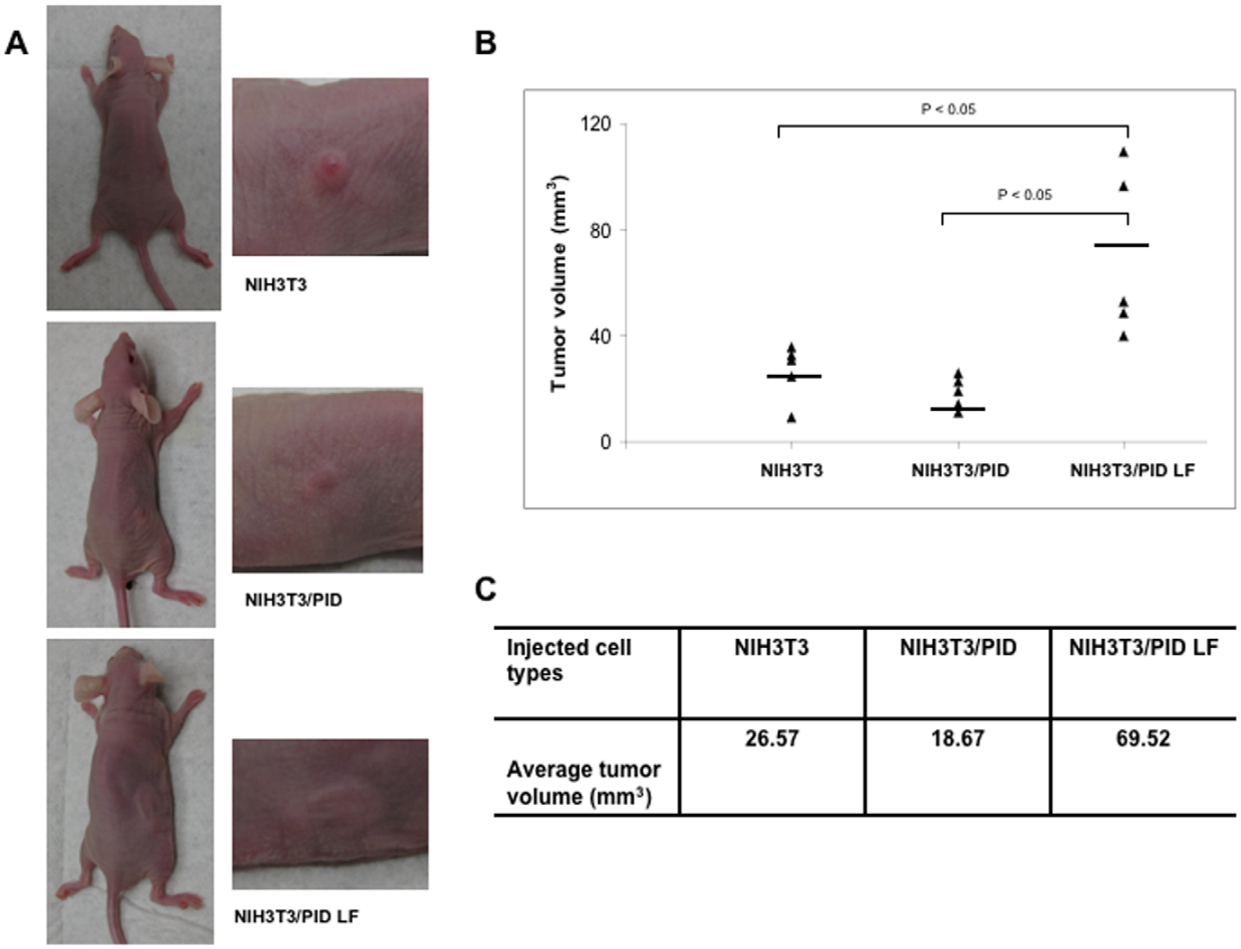
Effects of PID on tumor formation. Tumor formation was assessed by subcutaneously inoculating 1×10^6^ NIH-3T3 cells infected with empty vector (Vector) into left flanks and equal number of NIH-3T3 cells expressing PID or PID LF, respectively) into right flank of the same mice. Five mice were used for each cell line. Tumor diameters were measured every 3 days using caliper for 6 weeks following injection. (**A**) Representative photographs of nude mice with solid tumors (left panel). Enlarged images of boxed area in the left panel (right panel). (**B, C**) Tumor volume was calculated by the formula described in Material and Methods. Horizontal lines represent the average tumor diameter.

To assess the effects of Pak inhibition on NF2-related tumorigenicity, we injected nude mice only with ΔBB, ΔBB/PID, or ΔBB/PID LF cells. ΔBB cells developed substantial tumors by 6 weeks post-injection (average volume >200 mm^3^), similar to previous studies in which the Merlin BBA mutant was used [31], [32]. Tumors derived from ΔBB/PID cells were much smaller in size, with an average volume of 38 mm^3^ (Fig. 4). Interestingly, mice injected with ΔBB/PID LF cells developed tumors even larger (average volume ∼450 mm^3^) than those injected with ΔBB cells. Similar experiments were also carried out in NIH-3T3 cells lacking the ΔBB transgene (Fig. 5). In this latter case, only very small tumors were noted after 6 weeks (compare Fig. 4 with Fig. 5), but, as with the ΔBB xenograft, these masses were larger in cells expressing PID LF (Fig. 5).

**Figure 6:**
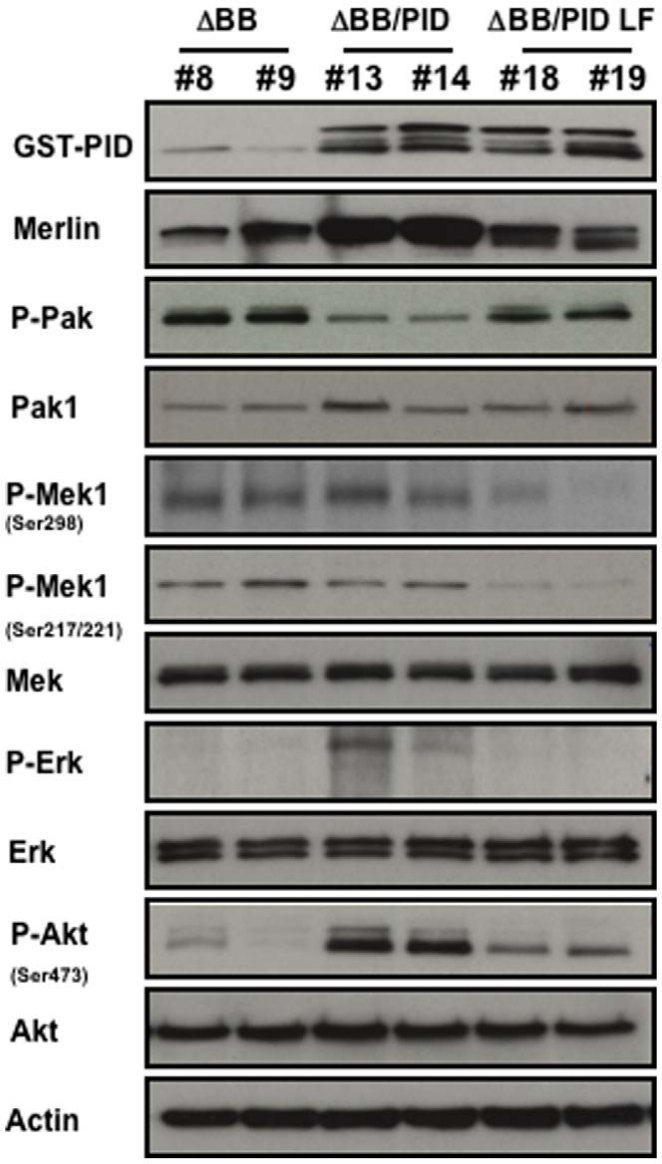
Activity of signaling pathways in tumor samples from xenografts. Tumor from six mice were resected and immediately lysed in a buffer containing 10 mM PBS pH 7.2, 1% Triton X-100, 0.1% SDS, 20% glycerol, plus complete protease and phosphatase inhibitor tablets (Roche). Lysates were separated by SDS-PAGE and incubated with the indicated antibodies.

**Figure 7:**
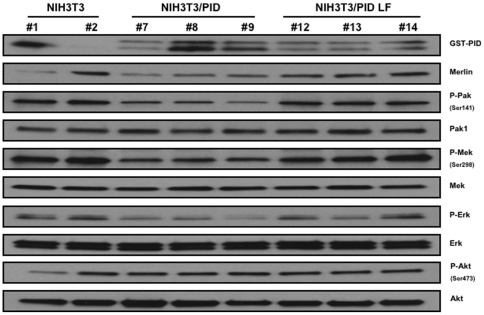
Effect of Pak inhibition on signaling in xenografts from NIH-3T3 cells. Tumors from eight mice were resected and immediately lysed in a buffer containing 10 mM PBS pH 7.2, 1% Triton X-100, 0.1% SDS, 20% glycerol, plus complete protease and phosphatase inhibitor tablets (Roche). Lysates were separated by SDS-PAGE and incubated with the indicated antibodies.

Lastly, we examined signaling activity in tumor lysates from these xenografts. Pak activity was inhibited in ΔBB/PID cells, indicating that this suppressor remained effective *in vivo*. Surprisingly, while Akt and Erk were not active in tumors derived from animals injected with ΔBB or ΔBB/PID LF cells, both Akt and Erk were activated in lysates from the small tumors that developed in mice injected with ΔBB/PID (Fig. 6). It is also of interest that, in the tumors from ΔBB/PID mice, Merlin expression (presumably exogenous Merlin ΔBB) was substantially elevated. These data show that PID expression strongly inhibited tumor formation in the NF2 xenograft model, suggesting that Group I Paks are required for transformation in cells that have lost Merlin function. Despite this requirement, loss of Pak function did not reduce Akt or Erk activity; on the contrary, it activated them. In the small tumors observed in NIH-3T3 xenografts, the effects of the PID on signaling were consistent with prior literature: PID expression decreased Pak activity, as well as the Mek phosphorylation at S298, Mek activity, and ERK activity (Fig. 7). In these small tumors, Merlin expression was not affected in a consistent way, nor was activation of Akt altered.

## Discussion

In this report, we have shown that the effects of a dominant acting Merlin protein can be largely reversed by inhibition of group I Paks. As has been noted previously, expression of a different “Blue Box” mutant of Merlin alters the actin cytoskeleton, promotes invasiveness, and confers the ability to form tumors in nude mice [31]. Given that Pak is activated in cells lacking functional Merlin [32] and that some of these aberrant phenotypes resemble those shown in cells with hyperactivation of Pak1, blockade of Pak might represent a reasonable therapeutic strategy in NF2.

The involvement of Akt and Erk in NF2 pathobiology is controversial. Some studies report elevated Akt and Erk in NF2-deficient cells [33], [34], [35], [36], whereas others report no correlation [37], [38]. Our results are consistent with these latter studies, in that cells lacking Merlin, whether grown *in vitro* or when recovered from xenograft tumors, displayed low basal Akt and Erk activity. Curiously, in the few small tumors that developed from ΔBB/PID xenografts, Akt and Erk activities were elevated (Fig. 6). The same was true for ΔBB/PID cells grown *in vitro* (data not shown). These activations may reflect an altered signaling strategy in the tumor cells, necessary to overcome loss of Pak activity due to PID expression.

Our studies also show that the ΔBB mutant of NF2, like another commonly studied “Blue Box” mutant, Merlin BBA, is in fact tumorigenic in mice. Whether this mutant acts in precisely the same manner as the better-studied BBA mutant is unclear, as these two mutants are reported to have different effects on cell adhesiveness and morphology. Despite these issues, it is clear that the ΔBB mutant has major effects on mouse cell morphology, invasiveness, and tumorigenicity, and that these changes are not accompanied by marked upregulation of Erk or Akt.

The data reported here are in general agreement with a previous study conducted by Yi *et al.*, in which Pak inhibition by shRNAs reduced tumor volume of dominant-negative NF2-expressing cells [32], but also have some intriguing differences. Interestingly, in the study by Yi *et al.*, escape from Pak knock-down occurred when expression of the Pak1 shRNA hairpin was silenced by methylation of the H1 promoter in the NF2 tumor cells. In our studies, which employed a peptide inhibitor of Pak rather than shRNA, we noted that the few, small tumors that arose in animals injected with ΔBB/PID cells did not shut off the PID transgene but did show significant increases in Merlin expression (Fig. 5). Because endogenous Merlin and the ΔBB Merlin mutant have similar mobility on SDS/PAGE, it was not possible to determine if the increased expression is due to endogenous or exogenous (ΔBB) Merlin, but we favor the idea that it represents ΔBB Merlin, especially in light of the lack of consistent increase in endogenous Merlin levels in xenograft tissues from cells expressing PID alone (Fig. 7). Therefore, we believe that the most likely explanation for these findings is that increased Merlin ΔBB expression was required for the tumor cells to escape inhibition consequent to diminished Pak function. Thus, both the results of our study and that of Yi *et al*. suggest that Pak activity is required for efficient tumorigenesis in cells that have lost Merlin function. In addition, our study shows that down-regulating Pak expression *per se* is not required for inhibiting tumor formation; inhibiting the catalytic activity of endogenous Pak is sufficient for these beneficial effects.

A number of peptide based reagents, such as PID and cell-penetrating peptides based on the Nck or PIX binding regions of Pak, have been used to effectively block Pak function in cells and *in vivo* [39], [40]. One note of caution raised by our studies is that the putative negative control for the PID, PID LF, appears to have gain-of-function effects in a variety of cell types. The PID LF mutant has been thought to represent a functionless, inert control for the PID, incapable of inhibiting Pak or binding to its partners such as the Fragile X protein [41]. Our results here should inject a note of caution in the use of this construct. Instead, one may consider small molecule inhibitors of Pak. Recently, a few specific small molecule inhibitors of group I Paks have also been described, including OSU03012, lambdaFL172 [42], and IPA-3 [43]. The latter of these three compounds has been shown to inhibit membrane ruffling and cell spreading in NF2^−/−^ schwannoma cells [44]. Interestingly, in these experiments, blockade of Pak with IPA-3 reduced Rac activity, suggesting that Pak acts upstream of Rac in these cells. Though Rac also activates Pak, such an idea is consistent with models which link Pak to further Rac activation via the Pak-bound guanine-nucleotide exchange factor, PIX [37], [45]. These results, like ours, support the notion that the Rac/Pak signaling axis is activated as a consequence of Merlin loss, and that these enzymes might provide useful targets for therapy in NF2.

## Materials and Methods

### Cell culture conditions and retroviral transductions

NIH-3T3 (obtained from American Type Culture Collection) cells were cultured in high-glucose DMEM supplemented with 10% fetal calf serum, 2 mM L-glutamine and 100 U/ml penicillin/streptomycin at 37°C in a humidified 5% CO_2_ incubator. The φNX packaging cell line (Orbigen) was transfected using Lipofectamine 2000 (Invitrogen). Viral supernatants were harvested 48 hr post-transfection and filtered. Cells were incubated with retroviral supernatant supplemented with 4 µg/ml polybrene for 4 hr at 37°C, and then were cultured in growth media for 48 hr for viral integration. Infected cells were selected with 2 µg/ml of puromycin or by flow cytometry for cells with green fluorescent protein (GFP).

### Expression Plasmids

A GST-PID (Pak1 amino acids 83–149) or Gst-PID L107F cDNA [27] was subcloned as a *Bgl*II/*Xho*I fragment into the retroviral expression vector pBMN-I-GFP (http://stanford.edu/group/nolan/plasmid_maps/pmaps.html), restricted with *Bam*HI/*Xho*I.

### Cell proliferation assay

10^4^ cells were plated in 96-well plates and 10 µl 3-[4,5-dimethylthiazol-2-yl]-2,5-diphenyl tetrazolium bromide (MTT) solution was added to each well to a final concentration of 0.5 mg/ml. The reaction was stopped after 4 hr at 37°C by adding 100 µl of solubilization solution (10% SDS in 0.01 M HCl) and the samples were analyzed at 595 nm on Perkin Elmer Envision plate reader. Triplicates were performed for each sample and medium alone was used as a blank, and experiments were performed on three occasions.

### Cell invasion assay

Matrigel invasion chamber (BD Biosciences) were rehydrated in serum-free DMEM medium for 2 hr and then placed in 0.75 ml of DMEM medium supplemented with 5% fetal calf serum. Cells at a density of 1.5×10^4^ suspended in 0.5 ml of DMEM, and seeded onto Matrigel chambers. Cells were allowed to invade for 12 or 24 hr and cells on the upper surface were gently removed with a cotton bud, and cells that had migrated through the 8 µm pores were fixed with 4% paraformaldehyde for 15 min and stained with 0.1% crystal violet for 15 min. Membranes were washed, removed and mounted on a glass slide, and the level of invasion was quantified by visual counting using a microscope with a 20X objective.

### Cell cycle analysis

Cell cycle profiles were analyzed using flow cytometry with propidium iodide staining. Cells were trypsinized and washed with PBS and fixed in 70% ethanol at −20°C. After fixation, cells were washed once with PBS and resuspended in 0.5 ml of a solution containing 1 M Tris-HCl pH 8.0, 0.1% Nonidet P-40, 10 mM NaCl, 50 µg/ml propidium iodide and 70 Kunitz units/ml RNase A. FACS analysis was performed using CELLQuest™ software (Becton Dickinson). A minimum of 10,000 events was collected per sample.

### Immunoblot analysis

Whole cell extracts were prepared by washing the cells in cold PBS and lysed in a buffer containing 50 mM Tris-HCl pH 7.5, 150 mM NaCl, 1% Nonidet P-40, and 0.25% sodium deoxycholate, 1 mM Na_3_VO_4_, and 1 mM NaF, plus complete protease inhibitor cocktail tablets (Roche). Protein concentrations were determined using bicinchoninic protein assay reagent according to manufacture's instructions (Pierce). Pak activation was stimulated by adding PDGF-BB (Sigma) at 5 ng/ml for 5 min to serum-starved cells. The antibodies used in this study include Pak1; Pak2; Mek1/2; phospho-MEK1 (Ser298); Erk1/2; phospho-Erk1/2 (Thr202/Tyr204); Akt; phospho-Akt (Thr308); Merlin (Cell Signaling Technology); phospho-Pak1–3 (Ser141; Invitrogen); GST (Santa Cruz Biotechnology); and β-actin (Sigma). All antibodies were used at a dilution of 1∶1,000, except for β-actin, which were diluted in 1∶20,000.

### Immunofluorescence

Cells were plated on glass coverslips in 6-well culture plates and fixed in 4% paraformaldehyde for 10 min, permeabilized with 0.2% Triton X-100 for 5 min and blocked with 1% BSA in PBS for 30 min. Filamentous actin and nuclei were visualized by staining with Rhodamine-phalloidin (Molecular Probe) and DAPI (Sigma), respectively. Images were observed and captured on an inverted phase/fluorescence microscope (Nikon TE300).

### Tumorigenicity assays

Cells were trypsinized, washed with PBS and resuspended at 10^7^ cells per ml in PBS. 2×10^6^ cells were injected subcutaneously into flanks of 5-week old nude mice (BALB/c *nu/nu*). Mice were monitored and tumor diameters were measured every 3 days using caliper for 6 weeks following injection. Tumor volume was calculated by the following formula: volume  = 0.5× (length) × (width)^2^. The mice were sacrificed and the tumors were resected for histological examination and immunoblot analysis. All animals were handled in strict accordance with good animal practice as defined by the relevant national and/or local animal welfare bodies, and all animal work was approved by the FCCC IACUC committee (Protocol 96-10), active 08/09 - 08/10.

### Data analysis

All experiments were performed at least three times. Results are reported as means ± SD. The significance of the data was determined by two-tailed, unpaired Student's *t*-test, and differences were considered statistically significant at *P*<0.05.

## Supporting Information

Figure S1 Delta BB Merlin expression in NIH-3T3 cells. Expression of Merlin from the experiment shown in Fig. 1B was quantitated using NIH Image J software.
(0.04 MB PDF)

Table S1 Raw cell invasion data for Fig. 2. Cell invasion studies were performed as described in Materials and Methods and in the legend to Fig. 2. Control insert  =  invasion in absence of Matrigel plug.
(0.05 MB DOC)

Table S2 Raw cell invasion data for Fig. S1. Cell invasion studies were performed as described in Materials and Methods and in the legend to Fig. 2. Control insert  =  invasion in absence of Matrigel plug.
(0.06 MB DOC)
