# Supplementary figures and images for: p21-Activated Kinases Are Required for Transformation in a Cell-Based Model of Neurofibromatosis Type 2
Source: PLoS One. 2010 Nov 2;5(11):e13791. doi: 10.1371/journal.pone.0013791 (PMC2970553; doi:10.1371/journal.pone.0013791)

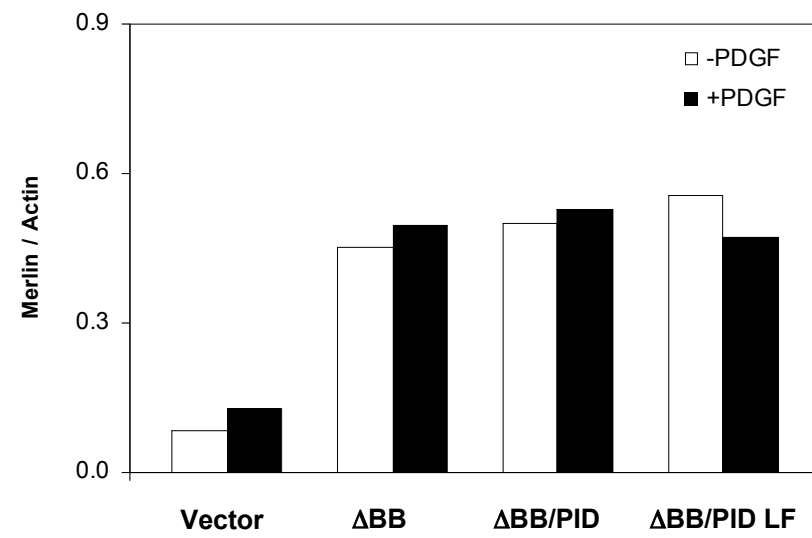

Supplement: Figure S1 — Delta BB Merlin expression in NIH-3T3 cells. Expression of Merlin from the experiment shown in Fig. 1B was quantitated using NIH Image J software. (0.04 MB PDF) [file pone.0013791.s001.pdf]
