# Supplementary material for: p21-Activated Kinases Are Required for Transformation in a Cell-Based Model of Neurofibromatosis Type 2
Source: PLoS One. 2010 Nov 2;5(11):e13791. doi: 10.1371/journal.pone.0013791 (PMC2970553; doi:10.1371/journal.pone.0013791)
Supplement: Table S1 — Raw cell invasion data for Fig. 2. Cell invasion studies were performed as described in Materials and Methods and in the legend to Fig. 2. Control insert = invasion in absence of Matrigel plug. (0.05 MB DOC) [file pone.0013791.s002.doc]

Experiment 1

|  | **Vector** | | | **ΔBB** | | | **ΔBB/PID** | | | **ΔBB/PID LF** | | |
| --- | --- | --- | --- | --- | --- | --- | --- | --- | --- | --- | --- | --- |
| **# cells invasion Matrigel Invasion Chambers** | 31 | 32 | 19 | 61 | 66 | 71 | 12 | 12 | 16 | 100 | 97 | 97 |
| **Mean** | 27.3 | | | 66 | | | 13.3 | | | 98 | | |
| **# cells migration (control insert)** | 149 | 132 | 156 | 195 | 191 | 197 | 203 | 222 | 210 | 169 | 181 | 175 |
| **Mean** | 145.7 | | | 194.3 | | | 211.7 | | | 175 | | |
| **Invasion** | 27.3/ 145.7 X 100 = 18.8% | | | 66 / 194.3 X 100 = 33.9% | | | 13.3/ 211.7 X 100 = 6.3% | | | 98 / 175 x 100 = 56.0% | | |

**Experiment** 2

|  | **Vector** | | | **ΔBB** | | | **ΔBB/PID** | | | **ΔBB/PID LF** | | |
| --- | --- | --- | --- | --- | --- | --- | --- | --- | --- | --- | --- | --- |
| **# cells invasion Matrigel Invasion Chambers** | 17 | 22 | 15 | 56 | 40 | 65 | 27 | 33 | 21 | 87 | 77 | 82 |
| **Mean** | 18 | | | 53.7 | | | 27 | | | 82 | | |
| **# cells migration (control insert)** | 105 | 98 | 107 | 153 | 172 | 139 | 178 | 199 | 210 | 167 | 176 | 203 |
| **Mean** | 103.3 | | | 154.7 | | | 195.7 | | | 182 | | |
| **Invasion** | 18/ 103.3 X 100 = 17.4% | | | 53.7 / 154.7 X 100 = 34.7% | | | 27/ 195.7 X 100 = 13.8% | | | 82 / 182 X 100 = 45.1% | | |

Experiment 3

|  | **Vector** | | | **ΔBB** | | | **ΔBB/PID** | | | **ΔBB/PID LF** | | |
| --- | --- | --- | --- | --- | --- | --- | --- | --- | --- | --- | --- | --- |
| **# cells invasion Matrigel Invasion Chambers** | 28 | 37 | 20 | 87 | 53 | 55 | 32 | 34 | 22 | 116 | 108 | 96 |
| **Mean** | 28.3 | | | 65 | | | 29.3 | | | 106.7 | | |
| **# cells migration (control insert)** | 139 | 156 | 222 | 219 | 200 | 191 | 222 | 219 | 200 | 162 | 188 | 186 |
| **Mean** | 172.3 | | | 203.3 | | | 213.7 | | | 178.7 | | |
| **Invasion** | 28.3/ 172.3 X 100 = 16.4% | | | 65/ 203.3 X 100 = 32.0% | | | 29.3 / 213.7 x 100 = 13.7% | | | 106.7/ 178.7 X 100 = 59.7% | | |

|  | **Vector** | **ΔBB** | **ΔBB/PID** | **ΔBB/PID LF** |
| --- | --- | --- | --- | --- |
| **Mean of invasion** | 17.6 | 33.6 | 11.3 | 53.6 |
| **SD** | 1.0 | 1.2 | 4.3 | 6.2 |
