# Supplementary material for: p21-Activated Kinases Are Required for Transformation in a Cell-Based Model of Neurofibromatosis Type 2
Source: PLoS One. 2010 Nov 2;5(11):e13791. doi: 10.1371/journal.pone.0013791 (PMC2970553; doi:10.1371/journal.pone.0013791)
Supplement: Table S2 — Raw cell invasion data for Fig. S1. Cell invasion studies were performed as described in Materials and Methods and in the legend to Fig. 2. Control insert = invasion in absence of Matrigel plug. (0.06 MB DOC) [file pone.0013791.s003.doc]

Experiment 1

|  | **NIH3T3** | | | **NIH3T3/PID** | | | **NIH3T3/PID LF** | | |
| --- | --- | --- | --- | --- | --- | --- | --- | --- | --- |
| **# cells invasion Matrigel Invasion Chambers** | 25 | 27 | 28 | 38 | 39 | 40 | 91 | 132 | 151 |
| **Mean** | 26.7 | | | 39 | | | 124.7 | | |
| **# cells migration (control insert)** | 125 | 112 | 112 | 188 | 192 | 196 | 213 | 201 | 258 |
| **Mean** | 116.3 | | | 188.7 | | | 224 | | |
| **Invasion** | 26.7/ 116.3 X 100 = 22.9% | | | 39/ 188.7 X 100 = 20.7% | | | 124.7/ 224 X 100 = 55.7% | | |

Experiment 2

|  | **NIH3T3** | | | **NIH3T3/PID** | | | **NIH3T3/PID LF** | | |
| --- | --- | --- | --- | --- | --- | --- | --- | --- | --- |
| **# cells invasion Matrigel Invasion Chambers** | 12 | 33 | 24 | 35 | 25 | 30 | 169 | 113 | 105 |
| **Mean** | 23 | | | 30 | | | 129 | | |
| **# cells migration (control insert)** | 82 | 99 | 77 | 207 | 131 | 163 | 213 | 288 | 218 |
| **Mean** | 86 | | | 167 | | | 239.7 | | |
| **Invasion** | 23/ 86 X 100 = 26.7% | | | 30/ 167 X 100 = 18.0% | | | 129/ 239.7 X 100 = 53.8% | | |

Experiment 3

|  | **NIH3T3** | | | **NIH3T3/PID** | | | **NIH3T3/PID LF** | | |
| --- | --- | --- | --- | --- | --- | --- | --- | --- | --- |
| **# cells invasion Matrigel Invasion Chambers** | 24 | 21 | 30 | 20 | 22 | 35 | 126 | 137 | 98 |
| **Mean** | 25 | | | 25.7 | | | 120.3 | | |
| **# cells migration (control insert)** | 71 | 65 | 80 | 188 | 135 | 103 | 189 | 220 | 196 |
| **Mean** | 72 | | | 142 | | | 201.7 | | |
| **Invasion** | 25/ 72 X 100 = 34.7% | | | 25.7/ 142 X 100 = 18.1% | | | 120.3/ 201.7 X 100 = 59.7% | | |

|  | **NIH3T3** | **NIH3T3/PID** | **NIH3T3/PID LF** |
| --- | --- | --- | --- |
| **Mean of invasion** | 28.9 | 18.9 | 56.4 |
| **SD** | 5.1 | 1.5 | 3.0 |

**Supplementary Table 2. Raw cell invasion data for Fig. S1.**
